# Genome Rearrangements Detected by SNP Microarrays in Individuals with Intellectual Disability Referred with Possible Williams Syndrome

**DOI:** 10.1371/journal.pone.0012349

**Published:** 2010-08-31

**Authors:** Ariel M. Pani, Holly H. Hobart, Colleen A. Morris, Carolyn B. Mervis, Patricia Bray-Ward, Kendra W. Kimberley, Cecilia M. Rios, Robin C. Clark, Maricela D. Gulbronson, Gordon C. Gowans, Ronald G. Gregg

**Affiliations:** 1 Department of Biochemistry and Molecular Biology, University of Louisville, Louisville, Kentucky, United States of America; 2 Pediatric Genetics Laboratory, University of Nevada School of Medicine, Las Vegas, Nevada, United States of America; 3 Department of Pediatrics, University of Nevada School of Medicine, Las Vegas, Nevada, United States of America; 4 Department of Psychological and Brain Sciences, University of Louisville, Louisville, Kentucky, United States of America; 5 Department of Pediatrics, Loma Linda University School of Medicine, Loma Linda, California, United States of America; 6 Department of Pediatrics, University of Louisville School of Medicine, Louisville, Kentucky, United States of America; Ohio State University Medical Center, United States of America

## Abstract

**Background:**

Intellectual disability (ID) affects 2–3% of the population and may occur with or without multiple congenital anomalies (MCA) or other medical conditions. Established genetic syndromes and visible chromosome abnormalities account for a substantial percentage of ID diagnoses, although for ∼50% the molecular etiology is unknown. Individuals with features suggestive of various syndromes but lacking their associated genetic anomalies pose a formidable clinical challenge. With the advent of microarray techniques, submicroscopic genome alterations not associated with known syndromes are emerging as a significant cause of ID and MCA.

**Methodology/Principal Findings:**

High-density SNP microarrays were used to determine genome wide copy number in 42 individuals: 7 with confirmed alterations in the WS region but atypical clinical phenotypes, 31 with ID and/or MCA, and 4 controls. One individual from the first group had the most telomeric gene in the WS critical region deleted along with 2 Mb of flanking sequence. A second person had the classic WS deletion and a rearrangement on chromosome 5p within the Cri du Chat syndrome (OMIM:123450) region. Six individuals from the ID/MCA group had large rearrangements (3 deletions, 3 duplications), one of whom had a large inversion associated with a deletion that was not detected by the SNP arrays.

**Conclusions/Significance:**

Combining SNP microarray analyses and qPCR allowed us to clone and sequence 21 deletion breakpoints in individuals with atypical deletions in the WS region and/or ID or MCA. Comparison of these breakpoints to databases of genomic variation revealed that 52% occurred in regions harboring structural variants in the general population. For two probands the genomic alterations were flanked by segmental duplications, which frequently mediate recurrent genome rearrangements; these may represent new genomic disorders. While SNP arrays and related technologies can identify potentially pathogenic deletions and duplications, obtaining sequence information from the breakpoints frequently provides additional information.

## Introduction

Many genetic diseases and disorders are caused by alteration of gene dosage due to duplication or deletion of large genomic regions [1]. More benign copy number variants (CNVs) are common, although they may contribute to normal individual variation and the occurrence of complex diseases in the general population [2], [3]. Many deletion/duplication abnormalities are known to cause intellectual disability (ID) and/or multiple congenital anomalies (MCA) as part of well-characterized genetic syndromes. While ID affects ∼2–3% of the population [4] and is the most common serious disability in children and young adults [5], an accurate diagnosis is possible in fewer than 50% of cases [4]–[6]. Visible chromosome aberrations, which constitute the majority of definitive diagnoses, are found in approximately 28% of individuals with ID [7]. Of the remaining cases, half are estimated to have an underlying genetic cause [5].

Many common genetic disorders, including Williams syndrome (WS, OMIM:194050) [8], Prader-Willi/Angelman syndromes [9] Smith-Magenis syndrome [10], and Charcot-Marie-Tooth disease type 1A/hereditary neuropathy with liability to pressure palsies [11], [12], are caused by submicroscopic chromosome abnormalities with recurring breakpoints. Clinical diagnoses are relatively straightforward for these syndromes because there are well-defined suites of clinical features and it is possible to rapidly test for the appropriate chromosome anomaly. Architectural features of the genome, most commonly low copy repeats (LCRs, also known as segmental duplications), are associated with deletion or duplication boundaries in these disorders and have been causally implicated in their characteristic genome rearrangements [1], [13]–[15]. Intra-chromosomal non-allelic homologous recombination (NAHR) between directly oriented LCRs causes deletions or duplications, while NAHR between inverted repeats leads to inversions [1]. With the advent of array comparative genomic hybridization (aCGH) and microarray techniques it is now possible to examine the genome of individuals with non-syndromic ID and MCA at even higher resolution. Such studies have identified putatively pathogenic genome rearrangements in 10–25% of otherwise undiagnosable ID cases [16]–[22]. Identification of affected genes in these cases may suggest targeted genetic tests in other probands with similar phenotypes.

The WS clinical phenotype includes elastin arteriopathy, developmental delay (DD) and/or ID, and a recognizable pattern of dysmorphic facial features [23]. Over the past several years we have studied the relation between phenotype and genotype in individuals with WS, which is characterized by a deletion of 7q11.23. During this time numerous individuals with an initial diagnosis of WS were referred to us whom on subsequent cytogenetic analyses were found not to have the typical 7q23.11 deletion. These individuals, who had ID and/or MCA, pose difficult challenges with respect to treatments and recurrence risk. In an attempt to ascertain the cause of the phenotypes in 31 such individuals we used SNP microarrays to determine genome wide copy number. We report that 6/31 individuals had large genome rearrangements, either deletions or duplications, which may be responsible for their clinical phenotypes. Two in particular were the result of alteration in regions flanked by LCRs, which may represent regions of genomic instability.

## Materials and Methods

### Participants

All participants were part of a 14-year study of genotype-phenotype relations in WS. Most of the probands in this report, 31 individuals with unidentified ID and or MCA, were referred to us with a clinical diagnosis of WS but subsequently tested negative for the expected 7q11.23 deletion using fluorescent in situ hybridization (FISH). In addition, samples from seven probands with cytogenetically confirmed chromosome 7 alterations and four individuals from the general population were used to validate our methods and ensure our analysis strategy could identify the expected alterations. Familial relationships were confirmed using the *GenePrint* GammaSTR kit (Promega, Madison, WI). All participants and/or their parents/guardians signed informed consent forms under protocols approved by the Institutional Review Boards of the University of Nevada School of Medicine and/or the University of Louisville.

### Cytogenetics

High-resolution cytogenetic analyses used standard methods including thymidine synchronization of the cultured cells and addition of ethidium bromide during metaphase harvest. FISH analyses were performed as described previously [24]. Probes were obtained through commercial sources (*MYCN* region, Vysis/Abbott, Des Plaines, IL) or generated from purified BACs. Observation was performed with a Zeiss Axioscop (Göttingen, Germany) and documented on a Metasystems (Altlussheim, Germany) imaging system. Image levels were adjusted in Photoshop CS2 (Adobe, San Jose, CA).

### SNP copy number determination

DNA was isolated from cultured lymphoblastoid cell lines (LBLs), fibroblasts, or peripheral blood lymphocytes (PBLs). DNA from PBLs was used whenever possible to exclude the possibility of cell line artifacts. RNA was isolated from LBLs using a Ribopure RNA isolation kit (Ambion, Austin, TX), and cDNA was synthesized using Superscript III reverse transcriptase and random primers (Invitrogen, Carlsbad, CA).

Genomewide SNP copy number was determined using the Affymetrix Human Mapping 500K SNP Array Set (Affymetrix, Santa Clara, CA) consisting of 250K *Sty*I and *Nsp*I subarrays containing probes for 238,304 and 262,264 SNPs, respectively. DNA was prepared for array analyses, and arrays were hybridized, washed, stained, and scanned following the manufacturer's protocol (Affymetrix, Santa Clara, CA). Genotypes were determined by Affymetrix GTYPE 4.0 software using the DM algorithm. CEL files were normalized and modeled in dChip using invariant set normalization and a perfect match/mismatch difference model [25]. Subarrays were normalized and modeled separately and subsequently combined for analyses. Copy number was inferred using median smoothing with a 7 SNP window and 10% trimming including all samples as references. Loss of heterozygosity was calculated by hidden Markov model considering haplotype with all samples considered to be references. MIAME compliant array data from this study have been uploaded to the Gene Expression Omnibus (GEO) database. Relevant data for the probands discussed in this manuscript will be submitted to the DECIPHER database.

CNVs were identified by statistical analysis of inferred copy number using Partek Genomics Suite 6.3 (Partek, St. Louis, MO). The significance of SNP copy number changes was determined using a 50 kb window and copy number thresholds of 1.5 and 2.4 for deletions and duplications, respectively. CNVs were detected using a minimum region size of 50 kb and *p*-value cutoff of 0.01. These parameters were selected to minimize false-positive results and were not suitable for the identification of small variants. Statistically identified regions were visualized in dChip to remove artifacts due to low SNP density and edited using raw copy number to more precisely refine endpoints. The boundaries of potentially pathogenic CNVs were confirmed by qPCR and cloned when possible.

Determining whether CNVs are likely to be pathogenic versus benign is one of the greatest difficulties currently facing clinical geneticists. We considered CNVs to be putatively benign if they are present as normal polymorphisms in the UCSC Genome Browser's structural variation track [26] and/or the Database of Genomic Variants [27], and/or were present in at least one of our general-population control samples. Novel CNVs that occurred in multiple probands with different clinical presentations were also considered to be normal polymorphisms. We chose to consider CNVs potentially pathogenic if they met one or more of the following criteria: (1) affected at least one gene whose haploinsufficiency or mutation is known to cause an abnormal phenotype based on the database of Online Mendelian Inheritance in Man [28]; (2) affected at least five Reference Sequence (RefSeq) genes whose copy numbers are not known to vary in the general population; (3) intersected a region associated with a known genetic disorder or Database of Chromosomal Imbalance and Phenotype in Humans using Ensembl Resources (DECIPHER) [29] feature. RefSeq genes are those annotated as part of the effort to provide a comprehensive list of all genes for all organisms (http://www.ncbi.nlm.nih.gov/RefSeq/index.html). In one case (9152) these criteria conflicted with a report of a CNV in the general population. This discrepancy was resolved by consideration of CNV credibility versus evidence for pathogenicity (see Discussion). Genes of unknown function that are strongly expressed in fetal and/or adult neural and/or cardiac tissues were considered potential candidates for developmental disorders with phenotypic features overlapping WS. Whenever possible, putative abnormalities were determined to be *de novo* by SNP array or qPCR.

### Cloning of deletion breakpoints

Microarray analyses allowed us to identify the location of molecular breakpoints to varying extents, which were largely determined by the SNP density on the arrays. In regions where the SNP density was low we designed qPCRs across the deleted region to narrow the interval to ∼40 kilobases (kb). To clone the deletion breakpoints we used one of several strategies. First, we designed PCR primers at 3–4 kb intervals between the nearest deleted and non-deleted region, on both sides of the deletion (Figure S1). PCR reactions using all combinations of forward and reverse primers then were analyzed. If any primer pair yielded PCR products, they were cloned and sequenced. If this strategy failed, we used either adaptor ligation based PCR walking [30] or inverse PCR to amplify junction fragments. For all identified junction fragments, PCRs were designed to confirm the junction position in genomic DNA. The sequence of the primers used and 100 bp of flanking DNA for each breakpoint are given in Table S1.

PCRs (20 µl) contained ∼100 ng of genomic DNA and AccuPrime *Taq* DNA Polymerase High Fidelity in buffer II (Invitrogen, Carlsbad, CA). DNA fragments were cloned into pCR-4-TOPO (Invitrogen Inc., Carlsbad, CA) and sequenced using the BigDye Terminator v3.1 kit (Applied Biosystems, Foster City, CA) on an ABI 3130xl Genetic Analyzer (Applied Biosystems, Foster City, CA). Sequences were mapped to physical positions on the February 2009 Human genome assembly, using the UCSC BLAST-Like Alignment Tool [31]. The BLAST 2 sequences program [32] was used to evaluate the regions flanking breakpoints for sequence similarity. Genome architecture at the breakpoints was examined using the segmental duplication [15] and RepeatMasker [33] tracks of the UCSC genome browser [26].

Quantitative PCR analyses were done using either TaqMan assays or Power SYBR Green PCR Master Mix (Applied Biosystems, Foster City, CA) and standard primers (Table S2) on an ABI 7900HT Real-Time PCR System (Applied Biosystems, Foster City, CA). All reactions (10 µl) contained 5 ng of DNA and were analyzed using conditions recommended by the manufacturer. Copy number using triplicate reactions was calculated by the instrument software using the ΔΔC_T_ method, with parental samples used as references whenever possible. Relative values for gene expression were determined using TaqMan assays for *GTF2I* (Hs00263393_m1) relative to 18S RNA (Hs01073657_m1) as recommended by the manufacturer (Applied Biosystems, Foster City, CA).

### Web Resources

BLAST 2 sequences, http://www.ncbi.nlm.nih.gov/blast/bl2seq/wblast2.cgi; Database of Genomic Variants, http://projects.tcag.ca/variation/; dChip, http://www.dchip.org; DECIPHER, http://decipher.sanger.ac.uk/; UCSC BLAT, http://genome.ucsc.edu/cgi-bin/hgBlat; UCSC Genome Browser, http://genome.ucsc.edu/cgi-bin/hgGateway. Reference Sequence (RefSeq), http://www.ncbi.nlm.nih.gov/RefSeq/index.html.

## Results

Genome copy number was determined using 500K SNP microarrays on 42 individuals. The analyzed samples fell into three groups. The first group contained 7 probands who had previously been identified with chromosome 7q11.23 alterations but whose clinical phenotypes suggested either deletion lengths not typical for WS or the possibility of additional genetic lesions. The second group consisted of 31 individuals who had ID/DD and/or MCA and had been previously diagnosed with WS but who did not have the characteristic 7q11.23 deletion. The third group consisted of 4 control individuals with normal phenotypes and karyotypes. Tables S3 and S4 summarize the molecular and clinical findings, respectively, of the individuals with genomic alterations described in detail below.

### Analyses of probands with chromosome 7 alterations

A major focus of our research effort has been to correlate phenotype and genotype in individuals with WS or other chromosome 7q11.23 alterations. Seven probands with cytogenetically confirmed chromosome 7 alterations were analyzed by microarrays: five probands with 7q11 deletions and two probands with 7q11 duplications. Two probands with WS and one proband with a duplication showed the expected deletion or duplication of the WS critical region, respectively, and are not discussed further. Of the remaining 4 probands, 3 had atypical deletions and one had a 7q11 duplication and a deletion on chromosome 1.

**Figure 1:**
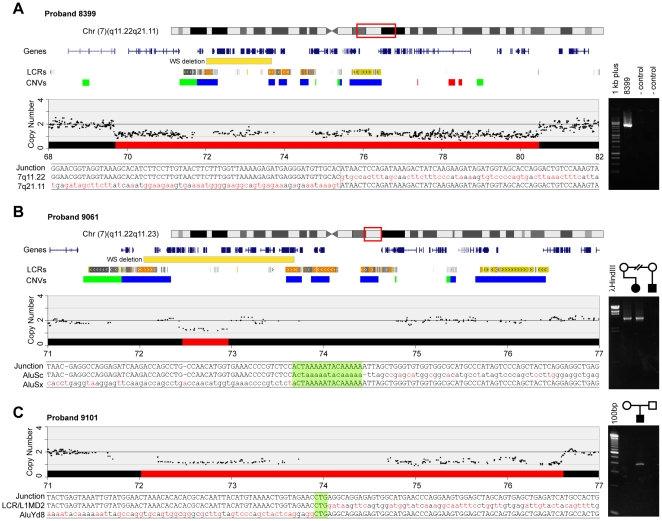
Breakpoint identification in individuals with deletions in the WS region and atypical phenotypes. Chromosome 7 ideograms showing the region of interest, including the typical WS deletion (yellow boxes) relevant to rearrangements in probands 8399, 9061, and 9101. The LCR and CNV tracks show the location of these features. The copy number track for each individual shows the copy number data from the arrays (black dots), analyzed as described in Methods. The red box on the ideogram represents the extent of the deletion. The sequence tracks show the sequence of the junction fragment aligned with the same region from the non-deleted chromosome. The sequence highlighted in green for probands 9061 and 9101 is present at both breakpoints so the actual break cannot be known. The gel images to the right show the validation of the junctions by PCR amplification of the junction fragment from genomic DNA. (**A**). Proband 8399 has a 10.8 Mb deletion that includes the entire WS typical region and includes 91 genes. The parents of 8399 were lost to follow up but the junction fragment was absent from two control samples. (**B**) Proband 9061 has a deletion of 503 kb affecting 13 genes, which includes only part of the WS critical region. The gel image to the right shows the junction fragment was inherited from her mother and was absent from an unaffected relative. (**C**) Proband 9101 has a 4.4 Mb deletion affecting 71 genes including the entire WS critical region. Chromosome ideogram is the same as that for 9061. The gel image shows this was a *de novo* deletion in the proband because the junction fragment was absent in both parents. A detailed list of affected genes in these three probands is provided in Figure S2.

Three of the probands with chromosome 7 deletions (8399, 9061, 9101) provide new insights into the nature of genome rearrangements and highlight the power of this approach to refine and discover new potentially pathogenic changes at the genome level. In all three cases we were able to clone the deletion breakpoints providing accurate information about which genes were deleted. Figure 1 shows the alignment of chromosome 7 ideograms indicating the extent of the deletions in these three individuals relative to the typical WS deletion. Below each schematic of the deletion is shown the sequence of the deletion junction.

Proband 8399 has WS and additional features including severe ID and a seizure disorder. The array analyses and subsequent cloning of the breakpoint showed this individual has a 10.8 Mb deletion (Figure 1A) which begins 1.2 Mb centromeric to and ends 3.5 Mb telomeric to the typical WS deletion. In total, this deletion removes 91 RefSeq genes (Figure S2).

Proband 9061 and four additional family members have been described previously (pedigree K3804 in ref. 24) and have an atypically small deletion within the WS region. They have normal intelligence but have deficits in visuospatial construction, which is characteristic of individuals with WS [34]. The SNP arrays refined the deletion end points sufficiently to allow us to clone a 4.2 kb fragment containing the deletion junction (Figure 1B). Sequence alignments indicated the deletion was 503 kb and includes 13 RefSeq genes (Figure S2). The deletion begins in *MLXIPL* and extends to and includes most of *LIMK1*. Haploinsufficiency of *LIMK1* is thought to be critical to the visuospatial construction deficits seen in individuals with WS and therefore is consistent with the phenotype in this proband.

**Figure 2:**
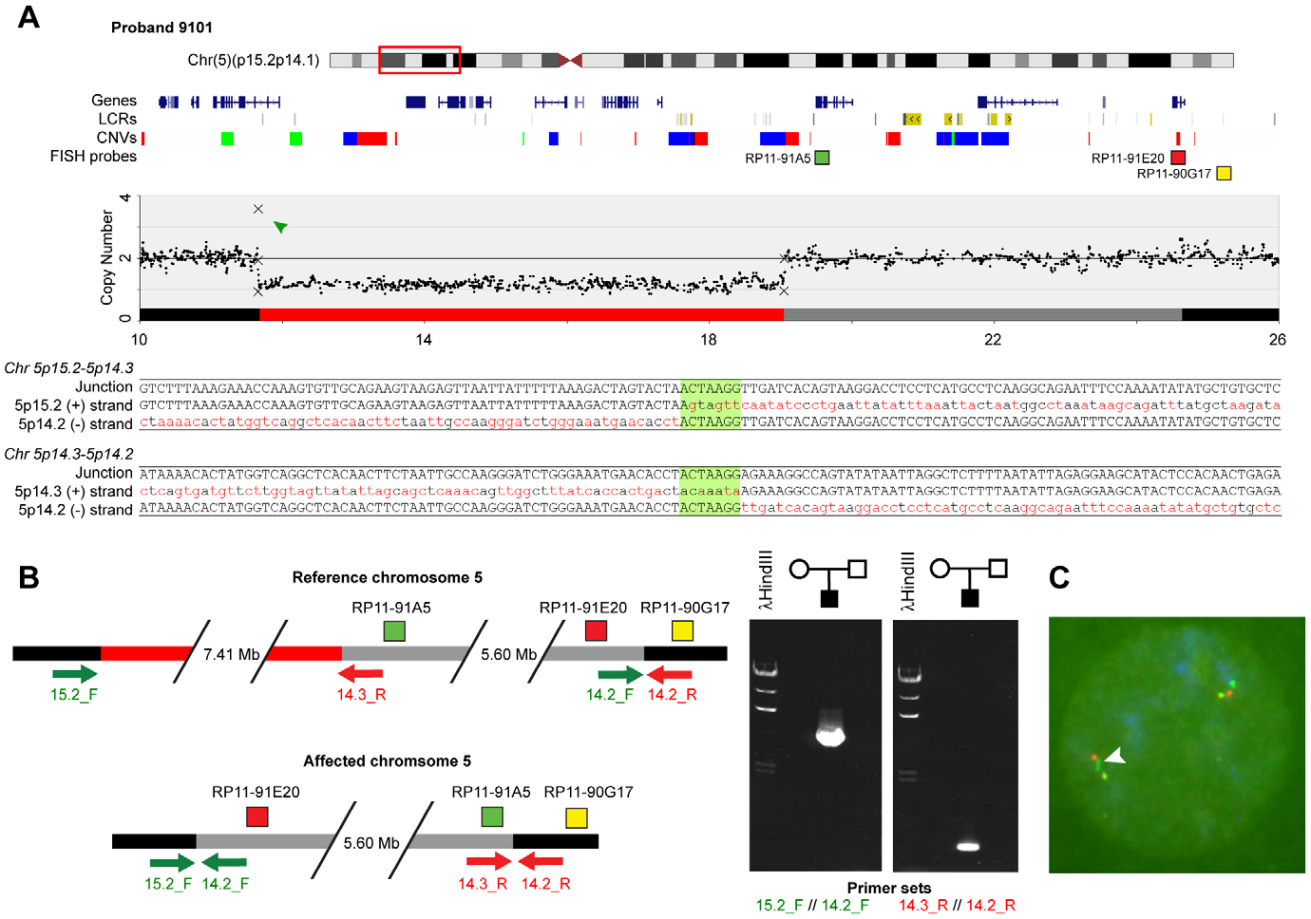
Determination of a complex rearrangement on chromosome 5p15.2-5p14.3 required sequencing deletion breakpoints. (**A**) Ideogram of chromosome 5 showing the 5p15.2‐5p14.3 deletion present in proband 9101. The copy number plot was derived from our microarray (black dots) and qPCR (Xs) results. The green arrowhead indicates an inherited CNV at the telomeric deletion breakpoint. Black and gray boxes depict regions of normal copy number, the red box indicates the extent of the deletion, and the gray box shows the inverted region. The sequence tracks show junction fragments along with genomic positions and orientations of flanking sequences. Eight base pairs of 5p14.2 sequence present at both breakpoints are highlighted in green. (**B**) Schematic of deletion and inversion including relative locations of PCR primers and FISH probes on reference and affected chromosomes 5. Telomeric is to the left. The gel image on the right shows amplification of the junction fragments present at the deletion/inversion breakpoints. The absence from both parents indicates this rearrangement occurred *de novo*. (**C**) Metaphase FISH analyses of cells from proband 9101, using BAC probes RP11‐91A5 (green), RP11‐91E20 (red), and RP11‐90G17 (yellow) confirming the presence of the inverted region of chromosome 5p14.5 (white arrowhead).

Proband 9101 has multiple congenital anomalies including supravalvar aortic stenosis and on cytogenetic analysis was found both to have the WS region deleted and also a translocation, t(7;11)(q21.1;p14), unrelated to the deletion and telomeric to the WS region. FISH analyses showed that the deletion involving the WS region extended further in the telomeric direction than does the typical WS deletion. Array analyses and cloning of the breakpoint indicated the deletion was approximately 4.4 Mb (Figure 1C) and affects 71 RefSeq genes (Figure S2). From the array analyses there was no indication that there were deletions or duplications associated with the translocation breakpoints t(7;11)(q21.1;p14) (data not shown). However, another large deletion of 7.38 Mb was detected on chromosome 5 that impacted 13 RefSeq genes (Figures 2A and S2). The reason this deletion was not detected in the original karyotyping is unclear but likely relates to the fact that it also involves a large inversion. We cloned the chromosome 5 breakpoint and the most parsimonious conclusion was that the deletion was also associated with an inversion, shown schematically in Figure 2B. PCR analyses of the predicted junction fragments supported this conclusion and showed the rearrangement occurred *de novo* in the proband. Finally, we used metaphase FISH with three BAC clones that based on the sequence predictions should be diagnostic of the inversion. These analyses show that one of the two chromosomes has the predicted inversion (Figure 2C). Analyses of the inversion breakpoints showed that the *CDH10* gene, located 5.6 Mb centromeric to the deletion, also was disrupted. The affected genes on chromosome 5 are all within the Cri du Chat syndrome region and likely contribute to the child's complex phenotype.

The fourth proband studied (9164) in this group was known to have a duplication in the WS region and initially was used as a control. However, we discovered a 1.46 Mb *de novo* deletion on 1q21.1 in this proband (Figure S2). This rearrangement has been previously reported as a genomic disorder with variable penetrance and has been found in both affected and unaffected individuals [35]. The clinical features in this proband are consistent with those of other individuals who have 7q11.23 duplications [36]–[38], indicating the deletion at 1q21.1 in this individual may not have any phenotypic effect.

### High frequency of genome copy number alterations in individuals with MCA and/or ID or DD

Over the past several years we have had many individuals referred to our study who carried a diagnosis of WS but who on subsequent clinical evaluation did not have WS. In general these individuals had non-syndromic MCA, and/or ID and/or DD. We used genome wide copy number analyses to screen 31 of these probands for potential genome abnormalities. Potentially pathogenic rearrangements were discovered in 6 probands. In these cases the alterations were confirmed using qPCR along with cloning breakpoints when possible. The location, size of alteration (deletion or duplication), number of RefSeq genes affected, and any features of interest at the breakpoints are given in Table S3. Three probands had deletions (including one mosaic deletion) and three had duplications that we hypothesize are responsible for their phenotypes, confirmation of which will require identification of additional individuals with similar alterations and phenotypes. The specific rearrangements for each of the 6 probands are described briefly below and in Table S3 part B. A clinical summary of each proband is given in Table S4, part B. In addition, our analyses identified 117 other regions of copy number variation (Table S5). Many of these have been previously reported in the general population, and none satisfy our criteria for potential pathogenicity.

**Figure 3:**
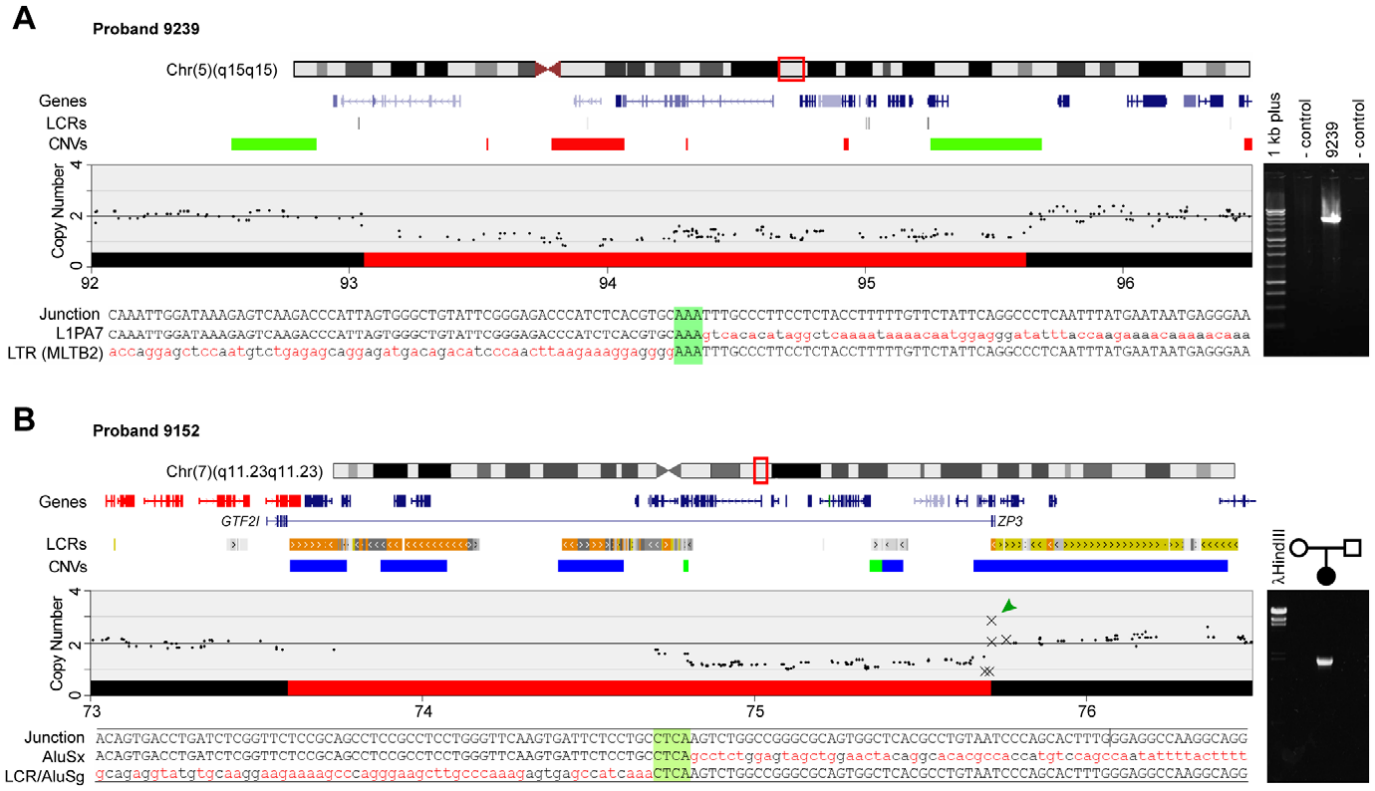
Deletion breakpoints on chromosome 5 and 7 associated with ID/MCA. Chromosome features for the region of interest are shown versus the physical position in Mb on the February 2009 human genome assembly. Red boxes on ideograms denote regions of detail. Gene and LCR tracks were adapted from the UCSC Genome Browser. CNV tracks show type and position as reported in the general population: red, loss; green, gain; blue, both. The copy number chart was derived from our SNP microarray (black dots) and qPCR (Xs) results. The red box on the copy number plots indicates the deletion. Sequence tracks from top to bottom show the junction fragment and the sequences flanking the centromeric and telomeric breakpoints. Regions of sequence identity at the breakpoints are highlighted in green. (**A**) Deletion of 2.57 Mb at 5q15 in proband 9239 that involves 15 genes. The gel image shows the presence of a PCR fragment amplified from the deletion junction in the proband but not in control samples (parental samples were not available). (**B**) Deletion of 2.13 Mb at 7q11.23 in proband 9152 that involves 34 genes. Genes shown in red are typically deleted in WS. This deletion includes a single gene, *GTF2I*, within the WS critical region. The *GTF2I-ZP3* fusion transcript resulting from the deletion is shown below the genes track. The green arrowhead shows the location of a CNV at the telomeric breakpoint inherited on the deleted chromosome 7. The gel image shows presence of a PCR fragment amplified from the deletion junction in genomic DNA from the proband, but not in either parental sample, indicating the deletion occurred *de novo*.

Proband 9239 has DD, microcephaly, and dysmorphic features. He has a 2.57 Mb deletion on chromosome 5q15. Sequencing of the breakpoint showed that 15 RefSeq genes were deleted (Figures 3A and S3). The centromeric breakpoint was within the *C5ORRF21* gene and the telomeric breakpoint just centromeric of *PCSK1*.

Proband 9152 has DD and mildly dysmorphic features. She has a *de novo* 2.13 Mb deletion at 7q11.23, with the centromeric breakpoint in the telomeric block of CNVs that give rise to WS (Figures 3B and S3). We were particularly interested in this deletion because of the possibility that *GTF2I*, which extends into the telomeric CNVs flanking the WS critical region, might be disrupted without other genes in the WS critical region being affected. *GTF2I* is a transcription factor, haploinsufficiency of which has been implicated in ID, visuospatial construction deficits, and/or personality characteristics associated with WS [24], [34], [38]. To evaluate whether *GTF2I* was disrupted we first examined its expression levels in LBLs from the proband, the proband's unaffected sibling, and seven unrelated individuals with known deletions that included *GTF2I*. The expression level of *GTF2I* in LBLs from proband 9152 was 49% of the sibling's and similar to that in the seven individuals with known deletions (data not shown), suggesting *GTF2I* may be disrupted in proband 9152. The SNP arrays we used had poor resolution in the CNVs that flank the WS region. However, the arrays localized the telomeric deletion breakpoint to within intron 6 of *ZP3* (Figure 3B). We hypothesized that if the deletion was in the *GTF2I* gene then a *GTF2I-ZP3* fusion transcript might be produced, which would allow us to precisely define the centromeric breakpoint. We used RT-PCR with primers in exon 2 of *GTF2I* and exon 7 of *ZP3* and amplified a 2.1 kb cDNA fragment from proband 9152. Sequence analysis showed it to be derived from a fusion of exons 2–9 and 11–12 of *GTF2I* and exon 7 of *ZP3*. This predicts the centromeric deletion breakpoint is within intron 12 of *GTF2I*, which is embedded in the CNVs that mediate the typical WS deletion. The fusion of *GTF2I* mRNA with *ZP3* is out of frame and is predicted to produce a truncated *GTF2I* protein that contains an additional 12 amino acids. This fragment of *GTF2I* contains the domain involved in dimer formation and could potentially act as a dominant negative, although its reduced expression level may decrease the likelihood of this outcome. In addition to *GTF2I* and *ZP3*, this child is haploinsufficient for 32 other RefSeq genes (Figures 3B and S2).

**Figure 4:**
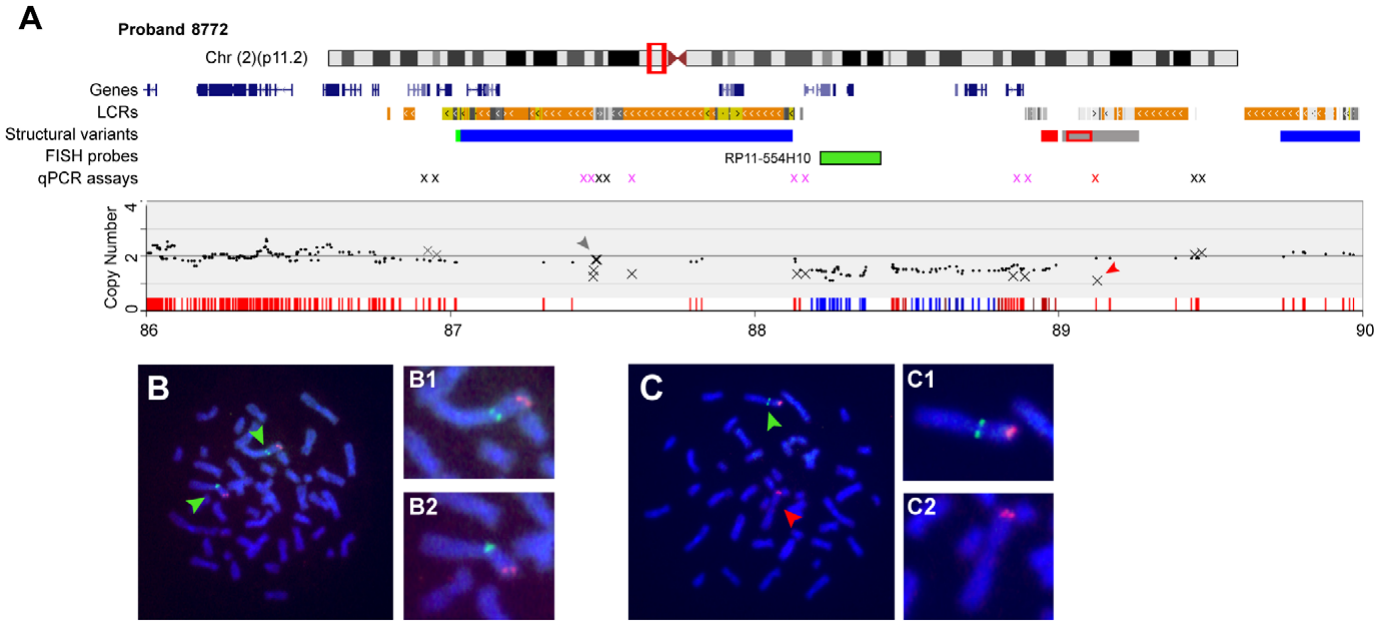
Identification of LCR mediated mosaic deletion using SNP arrays. (**A**) Chromosome features for the region of interest in proband 8772 are shown versus the physical position in Mb on the February 2009 human genome assembly. Red box on ideogram denotes region of detail. LCRs, structural variants, the FISH probes used and the location of qPCR assays also are shown. The copy number section shows the data for the SNP arrays (black dots) and qPCR (Xs) assays. The tick marks represent 2 copies (red) and 1.5 copies (blue). Note the paucity of SNPs in the LCRs and that the putative deletion ends in the LCRs. (**B, C**) metaphase FISH using BAC probe RP11-554H10 (green) and a BAC probe (red) located within *MYCN* at 2p24.3, which is distal to the region of interest. (**B**) FISH analyses show images from a normal cell from 8872. (**C**) FISH analyses showing a cell with one chromosome deleted for BAC RP11-554H10. (**B1, B2, C1, C2**) Detailed views of both chromosomes 2 in each cell. Note absence of hybridization to RP11-554H10 in C2.

One proband (8722), who was lost to follow-up, showed a SNP copy number of ∼1.5 for a 0.81 Mb region on chromosome 2p11.2 suggesting a mosaic deletion (Figures 4A and S3). To confirm mosaicism, we used FISH analyses with two BACs as probes, one in the putative deletion (RP11-554H10) and a second in an adjacent non-deleted region including *MYCN* (Figure 4B, C). In 79% of the cells two signals from each BAC were present indicating the cells were not deleted (Figure 4B). In the remaining 21% of cells one chromosome lacked a signal from the BAC within the putative deleted region (Figure 4C) confirming mosaicism. We were not able to clone a junction fragment from this proband because of the presence of LCRs at the deletion boundaries. However, qPCR data confirmed the deletion was at least 1.37 Mb and contained 14 RefSeq genes.

**Figure 5:**
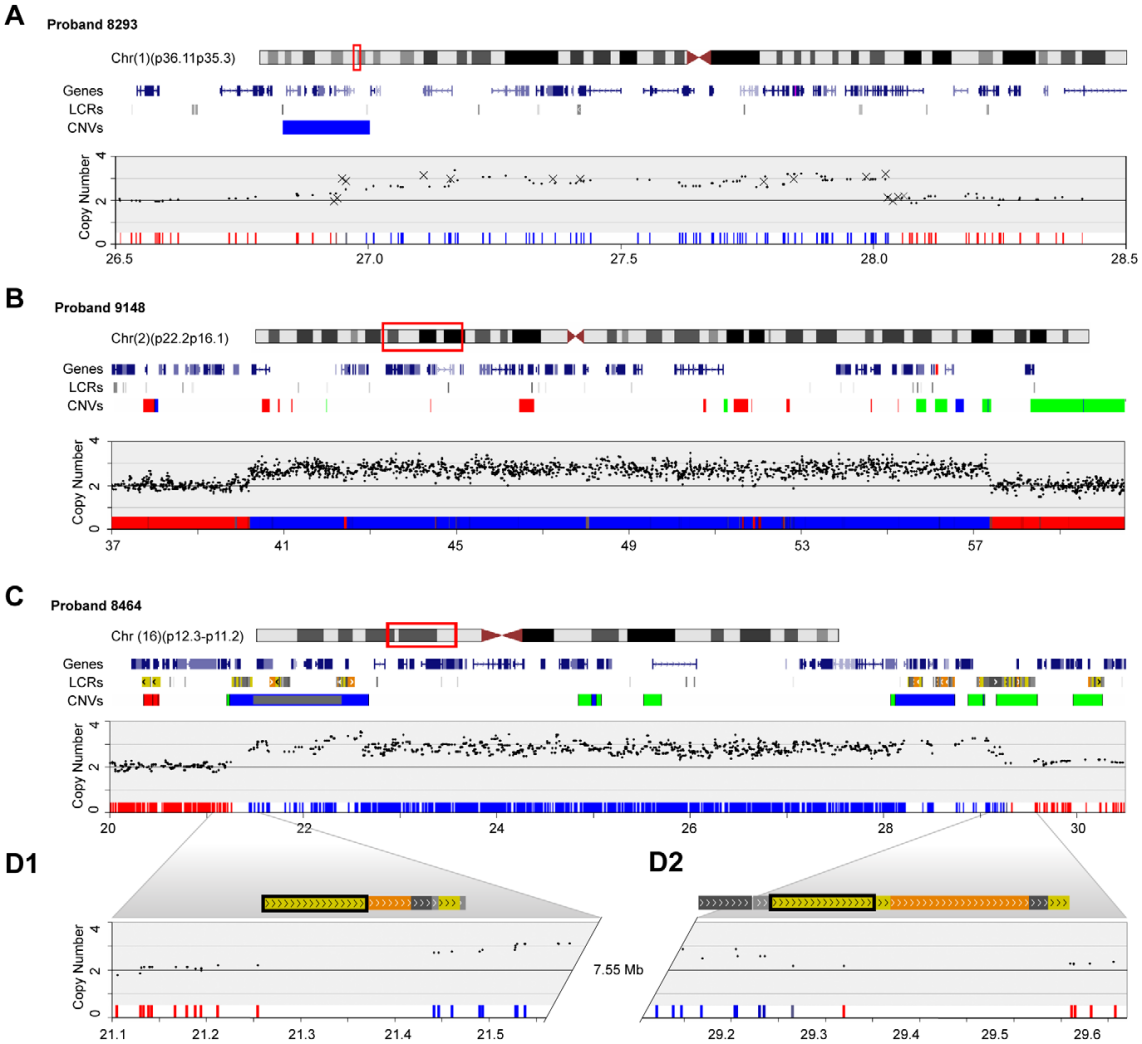
Duplications in probands with ID/MCA. Chromosome features for the regions of interest are shown versus physical position in Mb on the February 2009 human genome assembly. Red boxes on ideograms denote regions of detail. LCR and structural variant tracks were adapted from the UCSC Genome Browser. CNV and structural variation tracks show type and position of variants as reported in the general population: red, loss; green, gain; blue, both; grey, inversion. SNP copy number charts were derived from our microarray (black dots) and qPCR (Xs) results. Heat maps indicate *p*-values of observed copy number change for individual SNPs calculated using Partek Genomics Suite 6.3. Shading from blue to red represents probability from 0.0–1.0 of normal copy number. (**A**) Dup(1)(p36.11p35.3) in proband 8293. (**B**) *De novo* dup(2)(p22.1-2p16.1) in proband 9148. (**C**) *De novo* LCR-mediated dup(16)(p12.2-16p11.2) in proband 8464. (**D1**) and (**D2**) show detailed views of LCR architecture at the16p12.2 and 16p11.2 breakpoints, respectively. Directly oriented copies of UCSC segmental duplication 11963 are boxed in black.

The last group of probands with non-syndromic MCA and ID we describe had large duplications (Figures 5 and S4). Because of the nature of duplications cloning the end points was not feasible. However, the identification of these large rearrangements demonstrates the power of arrays to identify alterations not detected by standard cytogenetics. Proband 9148 has moderate ID plus dysmorphic features. She has a 17.16 Mb duplication at 2(p22.1p16.1) involving at least 74 RefSeq genes. Proband 8464 also has moderate ID and a range of other anomalies (Table S4). He has a 7.82 Mb duplication at 16(p12.2p11.2) involving 61 RefSeq genes. For these two cases we used FISH to demonstrate that the rearrangements were tandem duplications (data not shown). The final proband studied, 8293, has mild ID and a large number of other clinical signs (Table S4). He has a 1.1Mb duplication at 1(p36.11p35.3) involving at least 25 RefSeq genes. Large rearrangements of this region have not been previously reported, although the telomeric breakpoint is located in a CNV present in the general population [39].

The duplications in 9148 and 8464 have one or both breakpoints, respectively, in LCRs. The duplication in 8464 involves LCRs that are involved in mediating, or located near to, the boundaries of a reciprocal microdeletion syndrome with variable breakpoints [40]. Therefore, this duplication is likely to represent a new genomic syndrome. Confirmation will require ascertainment of additional individuals with similar duplications and phenotypes.

The three duplications described above contain large numbers of genes including many known to be important in development. As a consequence, we believe that these duplications are very likely responsible for the ID/MCA observed in the probands. However, confirmation of pathogenicity must await identification of additional cases with similar duplications.

## Discussion

High resolution cytogenetic analyses have been used clinically for many years and while enormously powerful; their resolution is usually restricted to detecting deletions and duplications of several megabases. Recently, several new technologies to examine genome copy number have been described and now are being used clinically. These include aCGH utilizing BAC clones and oligonucleotide arrays with dense whole genome coverage. In this report we describe the results from high density SNP array analyses of 38 individuals with unusual 7q11.23 alterations or with non-syndromic ID/DD and/or MCA. Consistent with other studies that have used this or similar technologies we found several individuals with large genome rearrangements that have not been described in the general population.

The use of microarrays yielded several benefits. In many cases the resolution of known cytogenetic abnormalities could be refined greatly and in several cases we were able to rapidly clone and characterize the deletion breakpoints. We found that, even in individuals previously characterized with high-resolution karyotypes and FISH for targeted regions, additional genome rearrangements were present. While this study is not the first to use this or similar technologies to characterize genome rearrangements, it is unique in that we characterized the extent of the deletions to very high resolution by cloning and sequencing several breakpoints.

These studies lead to important findings that need to be considered when interpreting data from arrays. The significance of CNVs in determining clinical phenotypes is difficult to determine. However they are often involved in the creation of genomic rearrangements, both benign and pathologic (see ref [14] for recent review). The consequence of these rearrangements with respect to their clinical relevance usually relies on knowledge of what constitutes normal verses pathogenic variation. Databases of structural variants and abnormalities [27], [29] are often used to determine whether an observed copy number change is potentially pathogenic. CNVs reported to be present in the general population are typically considered unlikely to be the causative mutation in individuals with abnormal phenotypes. However, the validity of this approach depends on the accuracy of the data in the databases. Many of the CNVs in public databases were computationally identified from genomic data and the alterations have not been validated using an independent method. This could lead to CNVs being considered to be normal population variants when in fact they cause clinically relevant phenotypes. We identified one such CNV, variation 3686 in the Database of Genomic Variants [27] at 7q11.23. Variation 3686 includes the complete coding region of *GTF2I* and several exons of *GTF2IRD1*. This CNV was reported to be present in 47 of 270 HapMap samples analyzed using BAC aCGH, but was not detected in the same samples when they were analyzed using 500K SNP microarrays [41]. Given that this deletion includes *GTF2I*, haploinsufficiency of which is pathogenic [24], [42], we believe that Variation 3686 is an artifact of the CGH array and/or the computational methods used to define/merge CNVs. The case of proband 9152 who has a CNV in this region provides a cautionary example of conflicts that may arise when using publicly available CNV data for interpretation of CNV data in a clinical setting.

Two of the chromosome rearrangements we identified here, mosaic del(2)(p11.2p11.2) and dup(16)(p12.2p11.2), are associated with LCRs. Both of these regions have been considered candidate loci for genome rearrangements in unexplained ID based on their segmental duplication architecture [43]. The 16p12.2-16p11.2 duplication discovered in proband 8464 is the reciprocal duplication of a recently described deletion disorder [39] and should be considered a putative genomic disorder pending identification of further cases with common breakpoints. Other duplications of 16p11-16p12 have been reported but not examined beyond the cytogenetic level [39], [40]. Identification of additional individuals with del(2)(p11.2p11.2) will be required to establish whether this rearrangement is in fact a recurrent finding in cases of non-syndromic MCA/ID.

Structural polymorphisms including deletions, duplications, and inversions are common in the general population and occur throughout the genome [3], [27], [41], [44]–[47]. It has been estimated that ∼12% of the human genome is likely to be copy number variable in the general population [48]. Although most structural variants do not appear to cause overt effects on phenotype, it is possible that some may predispose to pathogenic chromosome rearrangements. For instance, individuals carrying a common inversion polymorphism of the WS critical region [49] or copy number polymorphisms in the flanking LCRs [50] have increased likelihood of offspring with WS [51]. There are structural variants in the general population that co-localize with 11 of 21 (52%) of the breakpoints we have defined in this study. An elegant discussion of the importance of structural variation of the genome and the difficulties in CNV data interpretation has recently been published [52].

It is not unusual to discover that individuals with ID phenotypes have more than one significant genomic rearrangement, as seen in probands 9101 and 9164. In addition, large deletions and duplications are frequently complex in nature. Proband 9101, who carries a large deletion and an inversion that shared one of the deletion breakpoints, highlights this point. The inversion would not have been discovered had we not cloned the breakpoint, because there was no reason to suspect this defect. Further, the inversion inactivated an additional gene, which could be important for interpreting genotype-phenotype relations. The frequency of such complex rearrangements in individuals with deletions is currently unknown. In the very near future whole genome sequencing will become feasible from a cost perspective and such rearrangements will be readily detected. Until that time, care needs to be taken in interpreting CNV data, particularly using relatively low resolution methods.

Increasing the percentage of ID/MCA cases that can be rapidly and correctly diagnosed is a major goal for clinical genetics. We successfully used SNP microarrays to discover novel genome rearrangements in 6/31 (19%) of probands with non-syndromic ID/DD or MCA. Further, the ascertainment of two cases with unsuspected multiple chromosome rearrangements in a relatively small cohort suggests that this phenomenon may not be rare. The identification of additional rearrangements in some individuals on cloning the breakpoints, which were not detected by copy number measurements, indicates that care should be taken in genotype-phenotype correlations in the absence of sequence data. This concern will no doubt be eliminated as whole genome sequencing enters the clinic, increasing our understanding of the dynamics involved in sporadic chromosome rearrangements.

## Supporting Information

Table S1 Primers used for amplification of deletion junctions and sequences flanking breakpoints.
(0.05 MB PDF)

Table S2 Primer sequences for qPCR assays.
(0.08 MB PDF)

Table S3 Chromosome rearrangements characterized by SNP microarray.
(0.02 MB PDF)

Table S4 Clinical findings in probands with genome rearrangements.
(0.01 MB PDF)

Table S5 List of statistically identified CNVs.
(0.08 MB PDF)

Figure S1 Schematic representation of staggered primer method for identifying deletion junctions. Gene and LCR tracks are from the UCSC Genome Browser [26]. (A) SNP copy number results from 250K StyI array showing 503 kb deletion in proband 9061. Black dots on copy number track represent raw copy number, magenta triangles show copy number inferred by median smoothing using a 7 SNP window, and red line indicates a cutoff of 1.5 for defining deleted SNPs. (B) Detail of relative locations of PCR primers tested. The 4.1 kb fragment represents the junction fragment amplified and sequenced in this proband.
(0.16 MB TIF)

Figure S2 Schematic diagrams detailing genes deleted in probands. The red box on each diagram represents the affected region. The LCR-mediated, typical WS deleted region is shown in yellow on A and B. The RefSeq and sno/miRNA genes deleted for each proband are shown. Diagrams were adapted from UCSC Genome Browser [26]. (A,B) Genes affected on chromosome 7 including all or part of the WS typical region for probands (A) 8399, (B) 9164, 9061, 9101 and 9152. (C) Genes impacted by the deletion (red) and inversion (grey) on chromosome 5 of proband 9101. Note the disruption of CDH10 at the inversion junction. (D) Deletion on chromosome 1 of proband 9164 impacting 10 RefSeq genes.
(0.41 MB TIF)

Figure S3 Schematic diagrams showing genes affected by deletions in three probands with ID/MCA. Schematic diagrams of the affected chromosomes showing the location of the duplication on the February 2009 map. The green bars show the location of the duplicated region for the three probands (9239, 9152, 8771) discussed in the text.
(0.27 MB TIF)

Figure S4 Schematic diagrams showing genes affected by duplications in three probands with ID/MCA. Schematic diagrams of the affected chromosomes showing the location of the duplication on the February 2009 map. The green bars show the location of the duplicated region for the three probands (8293, 9148, 8464) discussed in the text.
(0.28 MB TIF)
