# Supplementary material for: Genome Rearrangements Detected by SNP Microarrays in Individuals with Intellectual Disability Referred with Possible Williams Syndrome
Source: PLoS One. 2010 Aug 31;5(8):e12349. doi: 10.1371/journal.pone.0012349 (PMC2930846; doi:10.1371/journal.pone.0012349)
Supplement: Table S1 — Primers used for amplification of deletion junctions and sequences flanking breakpoints. (0.05 MB PDF) [file pone.0012349.s001.pdf]

Table S1. Primers Used for Amplification of Deletion Junctions and Sequences Flanking Breakpoints

Case #	Chromosome Abnormality	Forward Primer (5'-3')	Reverse Primer (5'-3'	Breakpoint	Sequence	
8705		TGGTGGGGAAATCACATTATT	CATTAAAAGGCACAAGCTGTTAAA	Chr 7q22.1 +	AGGGGGGATTTGAACAAAGAAAAAACCCCGTGTGCATCGGAAAAGTGGCTGCTTATACATATCCTAAATCTAAAGGAAGTTTTGTTGTTGTTGTTGTTGT LCR(TTG)	
					/G/	
				Chr 7q31.31 +	AAGTGAAGGAAACATTAAGAGCAAAGTTCTTGAAATGAATTAATAATTGTCATATTTGATAAAGAGCAAGAAGTCCATTGTATTCTAAGCCTGGCACAGA	L2
8399	Del(7)(q11.22q21.11)*	CCAGAGGGCACATTGCTATT	CCCATTTACGTTTGCAATGA	Chr 7q11.22 +	CAATTCAGTTTTTGTTCTGGTTGATGTGGTTAGTGGAACGGTAGGTAAAGCACATCTTCCTTGTAACTTCTTTGGTTAAAAGAGATGAGGGATGTTGCAC/ Unique	
				Chr 7q21.11 +	ATAACTCCAGATAAAGACTATCAAGAAGATAGATGGTAGCACCAGGACTGTCCAAAGTAGACGTGAAAACCCAGATGATGGTTTCTTTTTTAGGCATATT	Unique
9101	Del(5)(p15.2p14.3)	TGCAGCCTTCAAAGCTTG	GTGGCCCACTGGAAGTAAGA	Chr 5p15.2 +	AGTAAGTCATTACTCATGCCTTCTGACTTGGGAATTTTGTCTTTAAAGAAACCAAAGTGTTGCAGAAGTAAGAGTTAATTATTTTTAAAGACTAGTACTA Unique	
	/ Inv(5)(p14.3p14.2)				/A /	
				Chr 5p14.2 -	ACTAAGGTTGATCACAGTAAGGACCTCCTCATGCCTCAAGGCAGAATTTCCAAAATATATGCTGTGCTCAGCTTTGGTCACTTTGTTGAATTGCATATAT	Unique
		CAGCAGAACGACGCCCCGCCGACA	GAGGCAAATGTATCCTTTGGTGAC	Chr 5p14.3 -	AAATGTTGAATAATTTTATATAGACACTTTAATAGAGTTCTCAGTTGTGGAGTATGCTTCCTCTAATATTAAAAGAGCCTAATTATATACTGGCCTTTCT/ Unique	
				Chr 5p14.2 +	CCTTAGTAGGTGTTCATTTCCCAGATCCCTTGGCAATTAGAAGTTGTGAGCCTGACCATAGTGTTTTATGTTTTTCATTTATCCCTTCTCATTGCCAAGG	Unique
9061	Del(7)(q11.23q11.23)a*	TCTTCCTCCGCTTCACATCT	TTCCCAATAAGCAGGTGAGG	Chr 7q11.23 +	GCTCACACCTGTAATCCCAGCACTTTGGGAGGCCTAGGTGGGTGAATAACGAGGCCAGGAGATCAAGACCAGCCTGCCAACATGGTGAAACCCCGTCTCC AluSc	
					/ACTAAAAATACAAAAA/	
				Chr 7q11.23 +	ATTAGCTGGGTGTGGTGGCGCATGCCCATAGTCCCAGCTACTCAGGAGGCTGAGACACAAGAATCACTTGAGCCCCGGAGGCGAAGGTTGTAGGGAGCTG	AluSx
9101	Del(7)(q11.23q11.23)b	CAGCAGAACGACGCCCCGCCGACAA	CTTGGCAGGCAATAGTTCACTT	Chr 7q11.23 +	ATTTAAAACCATTGTAAGATGAAGAGTCTATTTTTAGAGTACTGAGTAAATTGTATGGAACTAAACACACACGCACAATTACATGTAAAACTGGTAGAAC LCR(L1MD2)	
					/CTG/	
				Chr 7q11.23 +	AGGCAGGAGAGTGGCATGAACCCAGGAAGTGGAGCTAGCAGTGAGCTGAGATCATGCCACTGCACTCCAGCCTGGGTGACAGAGCGAGACTCCGTCTCAA	AluYd8
9239	Del(5)(q15q15)*	TCCCTGGCTAACAGATTGTTG	TCAGGAGACTGAATATTTTGAGAA	Chr 5q15 +	AAATGGGTTAAAAGCCACAATTAAAAGACACAGACTTGCAAATTGGATAAAGAGTCAAGACCCATTAGTGGGCTGTATTCGGGAGACCCATCTCACGTGC	L1PA7
					/AAA /	
				Chr 5q15 +	TTTGCCCTTCCTCTACCTTTTTGTTCTATTCAGGCCCTCAATTTATGAATAATGAGGGAAGATCTTCTTTACTCAGTCTCTGATTCAAATGCTAATCACT	LTR MLT2B2
9152	Del(7)(q11.23q11.23)c	GATGGGCAGTTGGACTACCT	ATCTGGGTCCTGCTCAGCTA	Chr 7q11.23 +	TTTTAAGACGGAGTCTTGCTCTGTTGCCCAGGCTGGAGTACAGTGACCTGATCTCGGTTCTCCGCAGCCTCCGCCTCCTGGGTTCAAGTGATTCTCCTGC AluSx	
					/CTCA /	
				Chr 7q11.23 +	AGTCTGGCCGGGCGCAGTGGCTCACGCCTGTAATCCCAGCACTTTGGGAGGCCAAGGCAGGTGGATCACAAGGTCAGGAGTTCAAGACCAGCCTGGCCAA	LCR(AluSg)

Sequences show 100bp flanking chromosome abnormalities and their observed orientations. Shared or inserted bases at junctions are shown on middle line. Breakpoints are indicated by /, inserted bases are shown in bold, regions of sequence identity are italicized, and sequence present at both breakpoints of chromosome 5p rearrangement are underlined. Type of repetitive sequence is shown to right. Forward primers for amplification of 9101 5p14.3 and 7q11.23 sequences are complementary to adaptor used for adaptor ligation mediated PCR walking [Padegimas & Reichert]. * denotes deletions cloned directly from SNP copy number data
