# Supplementary material for: Genome Rearrangements Detected by SNP Microarrays in Individuals with Intellectual Disability Referred with Possible Williams Syndrome
Source: PLoS One. 2010 Aug 31;5(8):e12349. doi: 10.1371/journal.pone.0012349 (PMC2930846; doi:10.1371/journal.pone.0012349)
Supplement: Table S2 — Primer sequences for qPCR assays. (0.08 MB PDF) [file pone.0012349.s002.pdf]

Table S2. Primer Sequences for qPCR Assays

Proband	Chromosome Abnormality	Primer Set	Forward Primer (5'-3')	Reverse Primer (5'-3')
**8293**	**Dup (1)(p36.11p35.3)**	qNUDC_1	GTAAGAACCGTCGCAAATATCGTG	TCCCCTACCACACACATTCG
		qNUDC_2	TGATAGGAGGGAAGGGGTAAC	CTGGCATTGTTTTCCTGTAGGT
		qNUDC_3	GATAGCTGTTTGCCTTTTCAGATG	CCTCTGCACTGCATATTTTCTCAT
		qSLC9A1_1	AGCCAGGGGTTTTCAAAATGTT	CCAGAATGTGCTGCCTTTTTAAGT
		qEYA3_1	CAAGGGGAAGGCTCCTCATT	CACCAGGCTTTAGAGCTTGATTT
		qEYA3-EX8	GTTTGAGATGCTGGCTACTGC	CACAAATGCCAGCCTGATATCT
		qEYA3-EX7	ATAATGTGCTGGGCTTGGC	TCCATCTCCAAGTCAACACAGT
**9148**	**Dup (2)(p22.1p16.1)**	q9148_1	ATGCACCCAGCCACTGCTAG	TTATCTGCTCCCAGCCAAGTG
		q9148_2	TGAAGCTCTGCTGCTTGTTTATG	GACTTCCCAGAGTTCCTGTCTTAT
		q9148_3	ATTTTCCCCAGGGACTTAGATTTT	CTGTCAAGTGGTAAAAGGGTTCAG
		q9148_4	CAGTGGGCAAATGGAGAAA	TGCTGAACAATATTCCATTGTGA
		q9148_5	GATTCCCACTCTTCCAAGTAACAT	AGGGTCCAAAGGGCTCAATAT
		q9148_6	CCATTCTCTGAATCCACAGAAAT	AAGTTTCCAGGGGTGTTCATAC
**8772**	**Mosaic Del (2)(p11.2p11.2)**	q8772_LCR1	ACCCCACTACATATCCCCAAGAT	AAATGACTTCCTAGGGAAGGAGC
		q8772_LCR3	TTTAATGTTGGTGTGAACAGTGG	CGTAGGGGCTACAAGATGCTATTT
		q8772_LCR5	CAGGGCCTCGAGCCACTT	ACCGTGCCTGTGTGCTGG
		q8772_LCR7	CCCCTGCCTGTGGCACCT	CCATGGAGAGGGCAGCTGG
		qCD8A_1	TGCGCACAACAGTATTGTGAC	TGACGGGAAACAAAACTAGGTAA
		qCD8B_1	GGTCTTTAGGATTTTGGGCAAATT	CCCTTGTGGAACCAAATGTATC
		qMGC4677_1	CGTAGGAAGGCTTGGGGTTAA	ACTTACATAAGGGCGGCATCC
		q8772telo_1	AGCTCCAGGGTTGCTTCTCAC	ATGCCTCTGCTTGGGTTGTC
		qKRCC1_1	GCAAAGGTCATTAATCCTCCAAT	AAATTGCTTTGATGGTGGAAATA
		qRPIA_2	AATTTTTAGTCACAAGCCTAGCTG	GACATCTGAAGTTGAAGCTATTGA
		qRPIA_3	AAAAGGCTTTAGATTTGTCATTCG	CCCAAAGCAAAAAGAGACTCAAA
		qIGKC_3	ATCAGCTGTGTTACTACGGAGTGC	CCATTTCCCCTCCCTCATCT
		qIGKC_1	TTGCCACACTCCCCTGAG	CTGCCCTCTCCAGTGTCATG
		qIGKC_2	TGAGCAACTTTCATAATTTCTCTT	AAGAGTTCCCTTGTGGCTAATAAA
**9101**	**Del (5)(p15.2p14.3)**	q9101-5p15_16	GCAAAAAATATAATGACCCCAAAAT	TGAATTGATCACACTGCTTAGAGA
		q9101-5p15_18	TCACCAAGCCTACGTAATGGA	TCTGGTATTTGGGATTTCCTATCT
		q9101-5p15_20	GTGTGGACTCATTTCCTGGAGATA	CTGAGACTTTAGCAGGCAACAGG
		q9101-5p15_11	AGAATTTTTCCCTGTTGAAACTCA	GCCAACTGTTTGAGCTGCTAAT
		q9101-5p15_10	GCTATGAAAGAAAAAGGCAGAGG	TGCATGTTTGTACCTTTGTTGTC
	**Del (7)(q11.23q11.23)**	q9101-7q11_1	TTTGCACCAAATGTAACGATAAA	CAAGTCATGCAAGAATCCTGTTA
		q9101-7q11_2	GCATACCTATAACAAAGGCCAAGA	TTAATGGACAACAAACCAGTGAA
		q9101-7q11_3	ATCAAATGCTATAAAAATCGTCAT	CTTTTGATCTATTCCCCTAAGGTT
		q9101-7q11_4	TAAAAGGAGGACAGAAACACATGG	TGTGGGTGTGTGTAAAATGGAGTA

Supplemental Table 1. Primer Sequences for qPCR Assays (continued)

	**Del (7)(q11.23q11.23)**	q9101-7q11_5	TTTATGATTTGGATTGCCAATTGA	AGGCAGATTTTTTTTCTGGCAGTA
		q9101-7q11_6	CAACAGTGCCATGTTCACTAAGA	ACGCCTACTCCATTTATCCAAA
		q9101-7q11_7	AATACAAATGCCACAAAGATGC	TAGTGTTGAGAGTGCTTTAAAAAA
		q9101-7q11_8	TGAGTACTACACAAGGGTTCATGC	ATGGGGCTTTCTACAAGGCATA
		q9101-7q11_9	GAGGTCCGAAGATGAGAAAGCA	CCAACCCAGCTGGATCATTTA
		q9101-7q11_10	GGAGCACAGGAAATCACTGTCA	TGGTGCTGTTGTTCCTGATGA
		q9101-7q11_11	GCCAAATCTTTGAGGGATTCT	TGTGGGAGATCCTTTATTCTCTG
		q9101-7q11_12	GGGGAGGGAAATGGATTTGAGT	CCTGGAATATCCTTTCCTTGTCCT
		q9101-7q11_13	ACCCACTGCCTGAATGTAAAA	TTAGGCCATGGATTTCTTTGAA
		q9101-7q11_14	GGGGCCAGAGATGTAATGTGT	CTTGGCAGGCAATAGTTCACTT
		q9101-7q11_15	TGGATAAATGGAGTAGGCGTACTT	AAAATGTCCTGGGCAGTAAATAAA
**9152**	**Del (7)(q11.23q11.23)**	q9152_1	ACAGGAATCTCAGGCATGTGGT	AGGGTCTGGCTTTTGGAAAC
		q9152_2	CCAGTCTGTTGAGTCCATGAGAA	AGGCGGGAATGTGGCAAG
		q9152_3	CCTCCACATGGAGCAGAGTTAAAG	ACAGGCAGTGACCCAGTGTT
		q9152_4	GTGTGAGGGAGGGCAGAATGT	GGCCCGTGGTATCATTCTCT
		q9152_5	TTGGTCATTATTTATGAAAGTGGT	AATAAGACCCTACCCTCCAAAAAT
		q9152_6	TGTGCCCTGATGCCTCTATT	CACCACAGGCAGACTGGA
		q9152_7	ACCTGGTTGCCCTTCAGGA	TACCCTCCATCAGACGCAGAGA
		q9152_8	ACGTGCCACTGCGGTTGT	TGTGATAAGGGGAGGCATTCTG
		q9152_9	GTAGCGGTCAGCATGGGTAT	CCCTCAACCCCCAGAGATT
		q9152_10	AGCCTTCCAACAGGTGAGGAG	TTGACAGATGTCAGCCGAGCC
		q9152_11	GGTTAAGCAGGAGGGGCTCT	TAGGATGACAGCAGATCTCCC
		qGTF2I_6	AGTTCGTTTATGATGAGTGGCAGTT	ACACAAGGTAAATAATTAGACAGTGATTGCA
		qGTF2I_7	TGGGAAGTTTGTATTTACCTGATAAGTTT[illegible]	CCCAGCACATCCAACTATTACCA
		qGTF2I_8	GGAACATATGGAAACATGCTTAATAATG[illegible]	CAGGGTCCTCACTTGTTTCTGAA
**8464**	**Dup (16)(p12.2p11.2)**	qCDR2	GCCGGTTTCTCCTGGTCAC	GCCAAGTGGAGGAGCTGAAGT
		qATXN2L	TACCCAGATGTTGAAGGGATTAAA	AAACATTTCAACAAGTCCAATCAC
		qZP2	TCTCTTACCTGGGTAGCTTTGC	TCAGTGAAGTTGGGTCCATTT
		qIGSF6	ACCTGTGTCCCCAGACGA	GCTGCTCATAGGCTCCATCA
		qCLN3	GGGCCTCGGTTCTTATGA	GGAGGGGAAGAAGAAGCAGA
