# Supplementary material for: Genome Rearrangements Detected by SNP Microarrays in Individuals with Intellectual Disability Referred with Possible Williams Syndrome
Source: PLoS One. 2010 Aug 31;5(8):e12349. doi: 10.1371/journal.pone.0012349 (PMC2930846; doi:10.1371/journal.pone.0012349)
Supplement: Table S3 — Chromosome rearrangements characterized by SNP microarray. (0.02 MB PDF) [file pone.0012349.s003.pdf]

Table S3. Chromosome rearrangements characterized by SNP microarray

Proband	Cytogenetic description	*de novo*	Detection method	Physical position	Size (Mb)	Genes affected	Structural variants at breakpoints	Sequence features at breakpoints	DNA source
**(A) Probands with known rearrangements**									
8399	Del (7)(q11.22q21.11)	Y	FISH	7:70243900-80836222*	10.79	91	-/-	Unique / Unique	LBL
9061	Del (7)(q11.23q11.23)	F	FISH	7:73022752-73525602*	0.50	13	-/-	AluSc / AluSx	PBL/LBL
9101	Del (5)(p15.2p14.3)	Y	500K SNP array	5:11612493-19014517*	7.38	13	+/+	TTTAAAN$_{36}$TTTAAAN$_{36}$TTTAAA / Uniqu	PBL/LBL
	Inv(5)(p14.3p14.2)	Y	Sequencing	5:19014518-24615888*	5.60	1	+/-	Unique / Unique	
	Del (7)(q11.23q11.23)	Y	FISH	7:72556311-76960517*	4.40	71	+/-	LCR(L1MD2) / AluYd8	
	t(7;11)(q21.1;p14)	Y	Karyotype	NA	NA	NA	NA	NA	
9164**	Del (1)(q21.1q21.1)	Y	500K SNP array	1:146101297-147808115	1.46	14	+/+	LCR/LCR	LBL
	Dup (7)(q11.23q11.23)	Y	aCGH, FISH	7:72755520-74289929	1.53	26	+/+	LCR/LCR	
**(B) Probands with unsuspected rearrangements**									
9239	Del (5)(q15q15)	?	500K SNP array	5:93028002-95601249*	2.57	15	-/+	L1PA7 / LTR MLT2B2	PBL
9152	Del (7)(q11.23q11.23)	Y	500K SNP array	7:74137882-76242236*	2.13	34	-/+	AluSx / LCR(AluSg)	PBL/LBL
8772	Mosaic Del (2)(p11.2p11.2)	Y	500K SNP array	2:87756166-89121981	1.37	14	+/+	LCR / LCR	LBL
8293	Dup (1)(p36.11p35.3)	?	500K SNP array	1:27264555-28339257	1.07	25	+/-	NA	LBL
9148	Dup (2)(p22.1p16.1)	Y	500K SNP array	2:40289097-57450235	17.16	74	-/+	NA	PBL
8464	Dup (16)(p12.2p11.2)	Y	500K SNP array	16:21534304-29358350	7.82	61	+/+	LCR / LCR	PBL/LBL/FIB

Physical positions are based on the Feb 2009 Human genome assembly [43, 44]; * denotes positional information based on sequence of breakpoints; The presence or absence of structural variants associated with breakpoints was determined using the UCSC Genome Browser [38] and Database of Genomic Variants [39] along with data generated by this study. ** The rearrangements in proband 9164 are stereotypical of 1q21.1 deletion and 7q11.23 duplication disorders, respectively. Abbreviations are: F, familial; FIB, fibroblast; LBL, lymphoblastoid cell line; PBL, peripheral blood
