# Supplementary material for: Genome Rearrangements Detected by SNP Microarrays in Individuals with Intellectual Disability Referred with Possible Williams Syndrome
Source: PLoS One. 2010 Aug 31;5(8):e12349. doi: 10.1371/journal.pone.0012349 (PMC2930846; doi:10.1371/journal.pone.0012349)
Supplement: Table S4 — Clinical findings in probands with genome rearrangements. (0.01 MB PDF) [file pone.0012349.s004.pdf]

Table S4. Clinical findings in probands with genome rearrangements

	Proband	Genetic Findings	Age (yrs)	Sex	Growth	OFC	Cognition	Behavior	Neurological	Craniofacial	Cardiac	Musculo-skeletal	Other
**(A) Probands with known rearrangements**													
	8399	Del (7)(q11.22q21.11)	7	F	NL	NL	Severe ID	ADHD	Seizure disorder, wide-based gait, hyper-reflexia LE	Ptosis, strabismus, periorbital fullness, flat mala, full cheeks, wide mouth, full lips	Pulmonic stenosis	Tight heel cords	Chronic otitis media, chronic constipation, Inguinal hernia
	9061	Del (7)(q11.23q11.23)	1	F	NL	NL	NA	NA	NL	NL	SVAS	NL	
	9101	Del (5)(p15.2p14.3), Inv(5)(p14.3p14.2), Del(7)(q11.23q11.23), t(7;11)(q21.1;p14)	0.5	M	Short stature	Micro	DD	NA	Central hypotonia, peripheral hypertonia	Stellate iris, short nose, broad nasal tip, flat midface	SVPS, bicuspid pulmonary valve	No joint laxity	GERD laryngomalacia, transient mild hypercalcemia
	9164	Del (1)(q21.1q21.1) Dup (7)(q11.23q11.23)	8	M	Short stature	Micro	Borderline IQ	ADHD	Mild dyskinesia, some symptoms of CAS	Facial asymmetry, upslanted palpebral fissures, long columnella, micrognathia, high arched palate	NL	mild brachydactyly	high pain tolerance, asthma, missing 3 permanent teeth, single palmar flexion crease
**(B) Probands with unsuspected rearrangements**													
	9239	Del (5)(q15q15)	2.5	M	NL	Micro	DD	ADHD	Hypotonia, persistent primitive reflexes, poor sleep	Short palpebral fissures, supraorbital fullness, short nose, anteverted nares, high arched palate, wide mouth with bowed upper lip, protruding lower lip	NL	NL	Inguinal hernia, Immunoglobin deficiency
	9152	Del (7)(q11.23q11.23)	3.75	F	NL	NL	DD	ADHD	Hypotonia	Mild facial asymmetry & right occipital flattening secondary to torticollis (resolved), stellate iris pattern, high arched palate, small squared pinna (length 5th%)	NL	NL	NA
	8772	Mosaic Del (2)(p11.2p11.2)	NA	F	NA	NA	NA	NA	NA	NA	NA	NA	Lost to follow-up
	8293	Dup (1)(p36.11p35.3)	20	M	NL	Macro	Mild ID	NL	Hypotonia	Upslanting palpebral fissures, high arched palate, low posterior hairline, small ears	NL	Large hands, kyphosis	Hoarse voice, constipation, esotropia
	9148	Dup (2)(p22.1p16.1)	15	F	Short stature	Micro	Moderate ID	ADD, OCD	Poor balance, poor fine motor skills	Short palpebral fissures, high arched palate, dental crowding, micrognathia	NL	Pectus carinatum, thin fingers	NA
	8464	Dup (16)(p12.2p11.2)	20	M	Short stature	NL	Mild ID	GAD	Hypotonia	Broad forehead, short philtrum, wide mouth, full lips	ASD	Joint laxity, tight hamstrings, pes planus	Chronic otitis media, Inguinal hernia

Abbreviations: M, male; F, female; NL, normal; NA, not available; OFC, orbitofrontal circumference; micro, microcephaly; macro, macrocephaly; ID, intellectual disability; DD, developmental delay; ADD, attention deficit disorder; ADHD, attention deficit hyperactivity disorder; GAD, generalized anxiety disorder; OCD, obsessive-compulsive disorder; CAS, childhood apraxia of speech; LE, lower extremities; SVAS, supravalvar aortic stenosis; SVPS, supravalvar pulmonic stenosis; ASD, atrial septal defect; GERD, Gastroesophageal reflux.
