# Supplementary material for: Genome Rearrangements Detected by SNP Microarrays in Individuals with Intellectual Disability Referred with Possible Williams Syndrome
Source: PLoS One. 2010 Aug 31;5(8):e12349. doi: 10.1371/journal.pone.0012349 (PMC2930846; doi:10.1371/journal.pone.0012349)
Supplement: Table S5 — List of statistically identified CNVs. (0.08 MB PDF) [file pone.0012349.s005.pdf]

Table S5. List of statistically identified CNVs

Chromosome	Start position	End position	Size (kb)	Type	Refseq genes	Includes exon	Previously reported CNV
1	26948697	28023399	1074.70	Gain	24	Y	N
1	63729851	63837565	107.71	Gain	2	Y	N
1	72258884	72374107	115.22	Loss	1	N	Y
1	97794409	98009752	215.34	Loss	1	Y	Y
1	102395713	102556188	160.48	Loss	0	N	Y
1	104304648	104515175	210.53	Gain	0	N	N
1	109401356	109466760	65.4	Loss	1	Y	N
1	143570846	144923027	1352.18	Loss	10	Y	N
1	216629257	216789404	160.15	Gain	3	Y	N
2	695636	912174	216.54	Gain	1	Y	Y
2	35734672	35997258	262.59	Loss	0	N	Y
2	40200748	57361886	17161.14	Gain	66	Y	N
2	87595428	88961243	1365.82	Mosaic loss	11	Y	N
2	88991630	89170082	178.45	Both	0	N	Y
2	89740700	89958350	217.65	Loss	0	N	Y
2	96206808	96258386	51.58	Gain	2	Y	N
2	97175459	97478937	303.48	Loss	2	Y	Y
2	110205175	110337721	132.55	Loss	2	Y	Y
3	19217997	19288605	70.61	Gain	1	Y	N
3	75535509	75627862	92.35	Loss	0	N	Y
3	147877590	148299630	422.04	Gain	0	N	N
3	169472632	169640335	167.7	Gain	0	N	N
4	59713	106981	47.27	Gain	2	Y	Y
4	93226702	93461993	235.29	Gain	0	N	N
4	93857899	93865520	7.62	Gain	1	N	N
5	752190	815465	63.28	Loss	0	N	Y
5	771021	815465	44.44	Gain	0	N	Y
5	11665493	19050273	7384.78	Loss	11	Y	N
5	29691322	29780415	89.09	Loss	0	N	N
5	63639566	63774328	134.76	Gain	0	N	N
5	93053757	95627006	2573.25	Loss	14	Y	N
6	12156242	12355596	199.35	Gain	1	Y	N
6	26936643	27056174	119.53	Loss	0	N	Y
6	31461668	31559455	97.79	Loss	2	Y	Y
6	168174708	168401562	226.85	Gain	3	Y	Y
7	7621327	7807665	186.34	Loss	1	Y	N
7	7964390	8301108	336.72	Gain	2	Y	N
7	31648115	31749521	101.41	Both	1	Y	N
7	38076360	38178211	101.85	Loss	1	Y	Y
7	38084338	38092618	8.28	Gain	1	Y	Y
7	53249866	53347121	97.26	Loss	0	N	Y
7	57216002	57453047	237.05	Gain	0	N	Y
7	64174103	64512781	338.68	Loss	1	Y	Y
7	69688551	80480873	10792.32	Loss	73	Y	N
7	72000962	76605168	4604.21	Loss	57	Y	N
7	72200171	73734580	1534.41	Both	25	Y	N
7	72467403	72970254	502.85	Loss	12	Y	N
7	73582532	75712811	2130.28	Loss	27	Y	Y*
7	85140643	85211420	70.78	Gain	0	N	Y
7	141528640	141941760	413.12	Loss	0	N	Y
8	6986630	8145978	1159.35	Gain	12	Y	Y
8	7222992	8106541	883.55	Loss	12	Y	Y
8	8164047	8182933	18.89	Both	0	N	Y
8	85484560	85528233	43.67	Loss	1	N	N
8	137757137	137931617	174.48	Loss	0	N	Y
9	251855	267989	16.13	Gain	1	Y	Y
9	11942128	12051706	109.58	Loss	0	N	Y
9	28497002	28667724	170.72	Loss	1	N	Y

9	31058070	31138008	79.94	Gain	0	N	N
9	40073195	43847424	3774.23	Gain	5	Y	Y
10	35046074	35120667	74.59	Loss	1	N	N
10	46363383	46557002	193.62	Loss	3	Y	Y
10	46363383	47162951	799.57	Gain	5	Y	Y
10	46397572	46557002	159.43	Gain	2	Y	Y
10	47062478	47143807	81.33	Gain	0	N	Y
10	59192085	59403275	211.19	Gain	0	N	Y
10	135158836	135264575	105.74	Gain	2	Y	Y
10	135176625	135238107	61.48	Loss	1	Y	Y
11	4085062	4133078	48.02	Gain	1	Y	Y
11	51049446	51318418	268.97	Gain	1	Y	N
11	80981836	81131219	149.38	Loss	0	N	Y
11	84140727	84214566	73.84	Loss	1	N	N
11	84211479	84214566	3.09	Gain	1	N	N
11	84211479	84233615	22.14	Loss	1	N	N
11	134060736	134367660	306.92	Gain	0	N	Y
12	7908745	8033723	124.98	Gain	2	Y	Y
12	31169298	31282170	112.87	Gain	1	Y	Y
12	31247316	31279220	31.9	Gain	1	Y	Y
12	72561457	72795885	234.43	Gain	0	N	N
12	72638877	72801220	162.34	Loss	0	N	N
13	42393686	42675338	281.65	Gain	2	Y	N
13	42447586	42632690	185.1	Gain	2	Y	N
13	42564370	42586916	22.55	Loss	1	Y	N
13	90860332	91099862	239.53	Loss	1	Y	N
13	91085865	91089064	3.2	Loss	1	N	N
14	19272965	19492423	219.46	Gain	6	Y	Y
14	19309319	19492423	183.1	Loss	5	Y	Y
14	21338108	22045568	707.46	Loss	0	N	Y
14	21929710	22016220	86.51	Loss	0	N	Y
14	44824833	44924986	100.15	Loss	0	N	Y
14	51669842	51846098	176.26	Gain	1	Y	N
14	105149735	106356482	1206.75	Loss	0	N	Y
14	105149735	105170875	21.14	Gain	0	N	N
14	105829129	106250892	421.76	Loss	0	N	N
14	105988336	106044701	56.37	Loss	0	N	Y
14	106223861	106246130	22.27	Gain	0	N	Y
15	18711364	20329239	1617.88	Gain	6	Y	Y
15	19821421	20335459	514.04	Loss	4	Y	Y
15	19827281	20329239	501.96	Gain	4	Y	Y
15	20329239	20686315	357.08	Gain	4	Y	Y
15	22140119	22299861	159.74	Gain	0	N	Y
15	28393128	28864940	471.81	Loss	2	Y	Y
15	56927621	57051456	123.84	Loss	2	Y	N
15	82618970	82889733	270.76	Loss	0	N	Y
15	82878742	82892291	13.55	Gain	0	N	Y
15	99536223	99708384	172.16	Gain	4	Y	Y
16	18152877	18728692	575.82	Gain	4	Y	Y
16	21441805	29265851	7824.05	Gain	69	Y	N
16	34307201	34614572	307.37	Gain	0	N	Y
16	85527980	85609265	81.29	Loss	0	N	Y
17	1130370	1900332	769.96	Gain	19	Y	N
17	20549240	20575451	26.21	Gain	0	N	Y
17	41006823	41011471	4.65	Gain	0	N	Y
17	41006823	41015120	8.3	Loss	0	N	Y
17	41006823	41015664	8.84	Gain	0	N	Y
17	41521621	41719833	198.21	Gain	1	Y	Y
17	41536766	41708649	171.88	Loss	1	Y	Y
18	17131449	17144202	12.75	Loss	0	N	N
18	64126718	64251503	124.79	Gain	0	N	Y

18	72892105	73087926	195.82	Gain	1	Y	N
19	56977641	57296437	318.8	Gain	8	Y	Y
20	14653888	14856035	202.15	Loss	1	N	Y
20	14729684	14771472	41.79	Loss	1	N	Y
21	24608865	24705724	96.86	Gain	0	N	N
22	21053688	21546762	493.07	Loss	0	Y	Y
22	21387732	21546762	159.03	Loss	0	N	Y
22	24043866	24255338	211.47	Gain	1	Y	Y
22	24113145	24240404	127.26	Gain	0	N	Y
22	31337616	31417223	79.61	Gain	1	N	N

Physical positions based on the May 2004 human genome assembly. The Database of Genomic Variants [39] and UCSC Genome Browser [38] were used to determine whether variants were previously reported. Rearrangements discussed in the manusript are shown in red. *We believe that an earlier report of a CNV including the deletion in 9152 was a false-positive (see general discussion)
