# Supplementary figures and images for: Genome Rearrangements Detected by SNP Microarrays in Individuals with Intellectual Disability Referred with Possible Williams Syndrome
Source: PLoS One. 2010 Aug 31;5(8):e12349. doi: 10.1371/journal.pone.0012349 (PMC2930846; doi:10.1371/journal.pone.0012349)

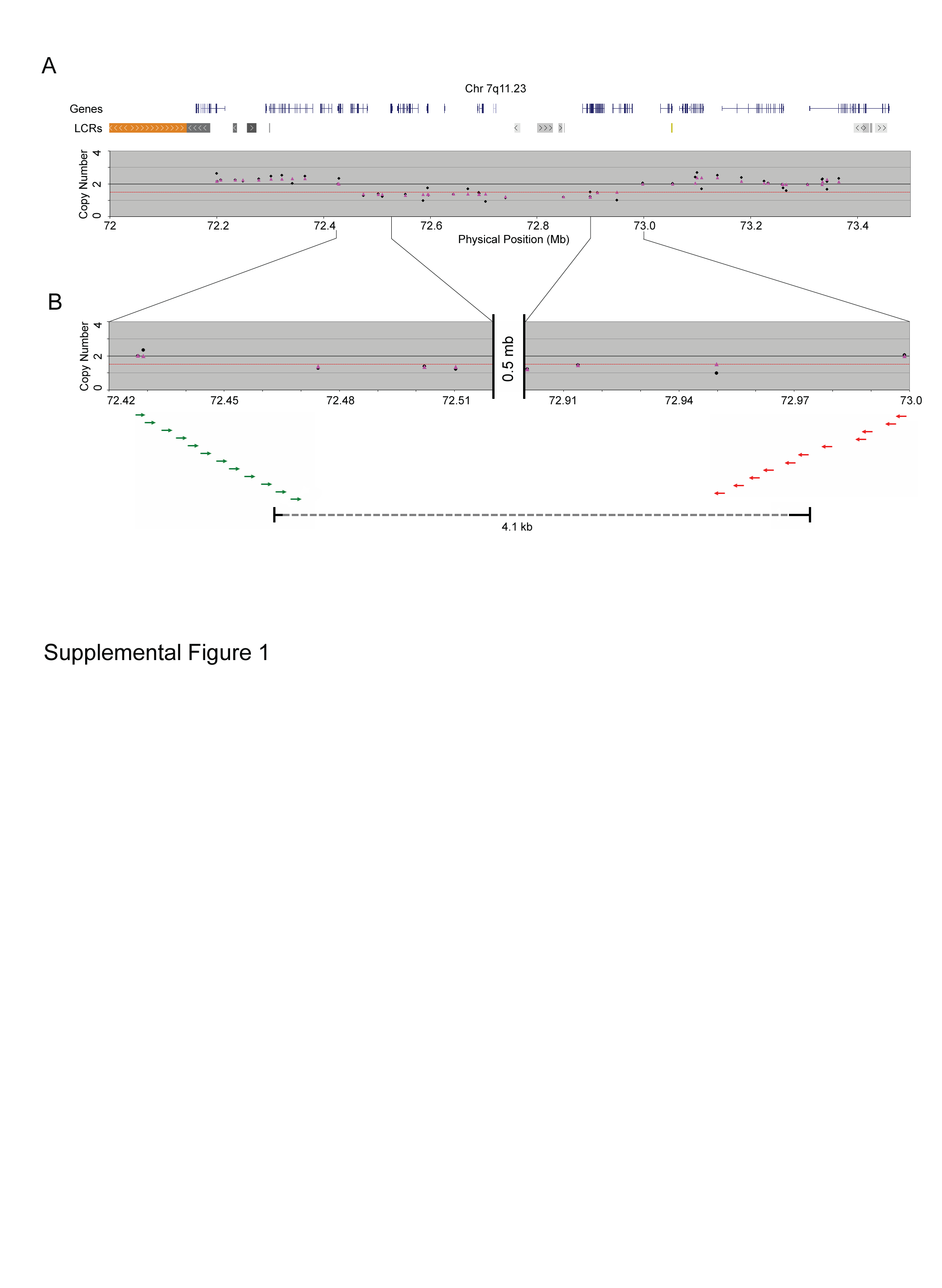

Supplement: Figure S1 — Schematic representation of staggered primer method for identifying deletion junctions. Gene and LCR tracks are from the UCSC Genome Browser [26]. (A) SNP copy number results from 250K StyI array showing 503 kb deletion in proband 9061. Black dots on copy number track represent raw copy number, magenta triangles show copy number inferred by median smoothing using a 7 SNP window, and red line indicates a cutoff of 1.5 for defining deleted SNPs. (B) Detail of relative locations of PCR primers tested. The 4.1 kb fragment represents the junction fragment amplified and sequenced in this proband. (0.16 MB TIF) [file pone.0012349.s006.tif]

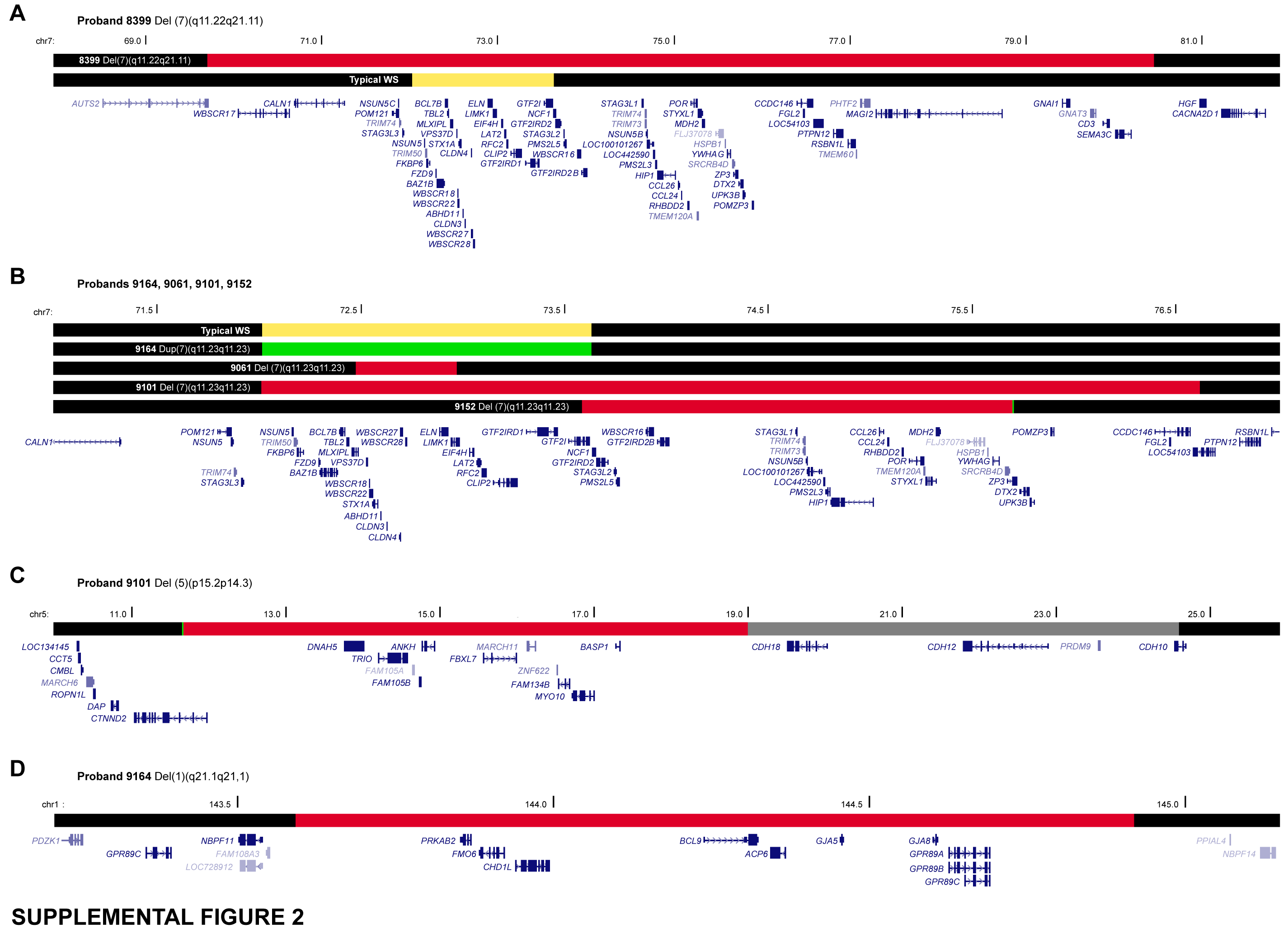

Supplement: Figure S2 — Schematic diagrams detailing genes deleted in probands. The red box on each diagram represents the affected region. The LCR-mediated, typical WS deleted region is shown in yellow on A and B. The RefSeq and sno/miRNA genes deleted for each proband are shown. Diagrams were adapted from UCSC Genome Browser [26]. (A,B) Genes affected on chromosome 7 including all or part of the WS typical region for probands (A) 8399, (B) 9164, 9061, 9101 and 9152. (C) Genes impacted by the deletion (red) and inversion (grey) on chromosome 5 of proband 9101. Note the disruption of CDH10 at the inversion junction. (D) Deletion on chromosome 1 of proband 9164 impacting 10 RefSeq genes. (0.41 MB TIF) [file pone.0012349.s007.tif]

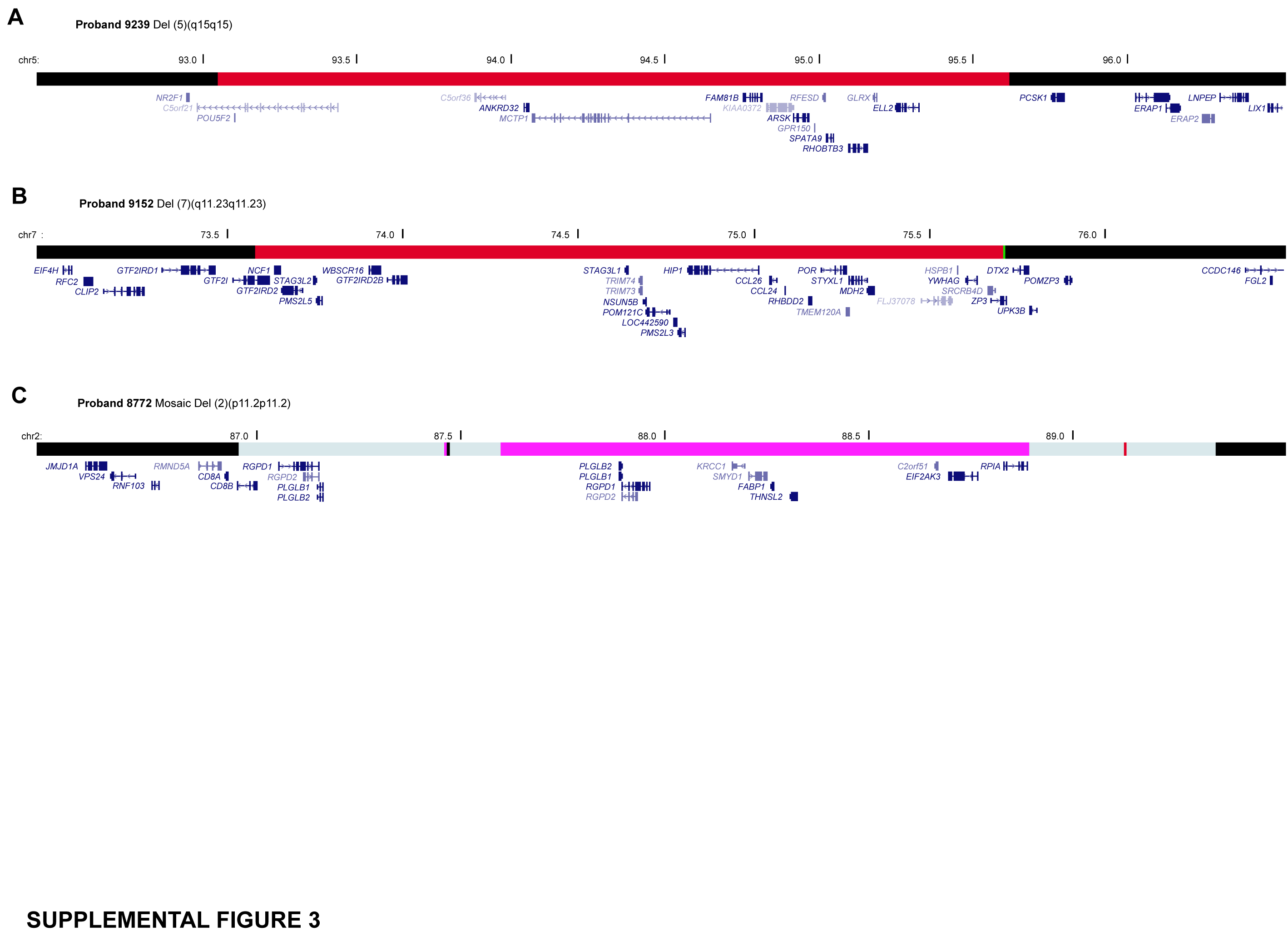

Supplement: Figure S3 — Schematic diagrams showing genes affected by deletions in three probands with ID/MCA. Schematic diagrams of the affected chromosomes showing the location of the duplication on the February 2009 map. The green bars show the location of the duplicated region for the three probands (9239, 9152, 8771) discussed in the text. (0.27 MB TIF) [file pone.0012349.s008.tif]

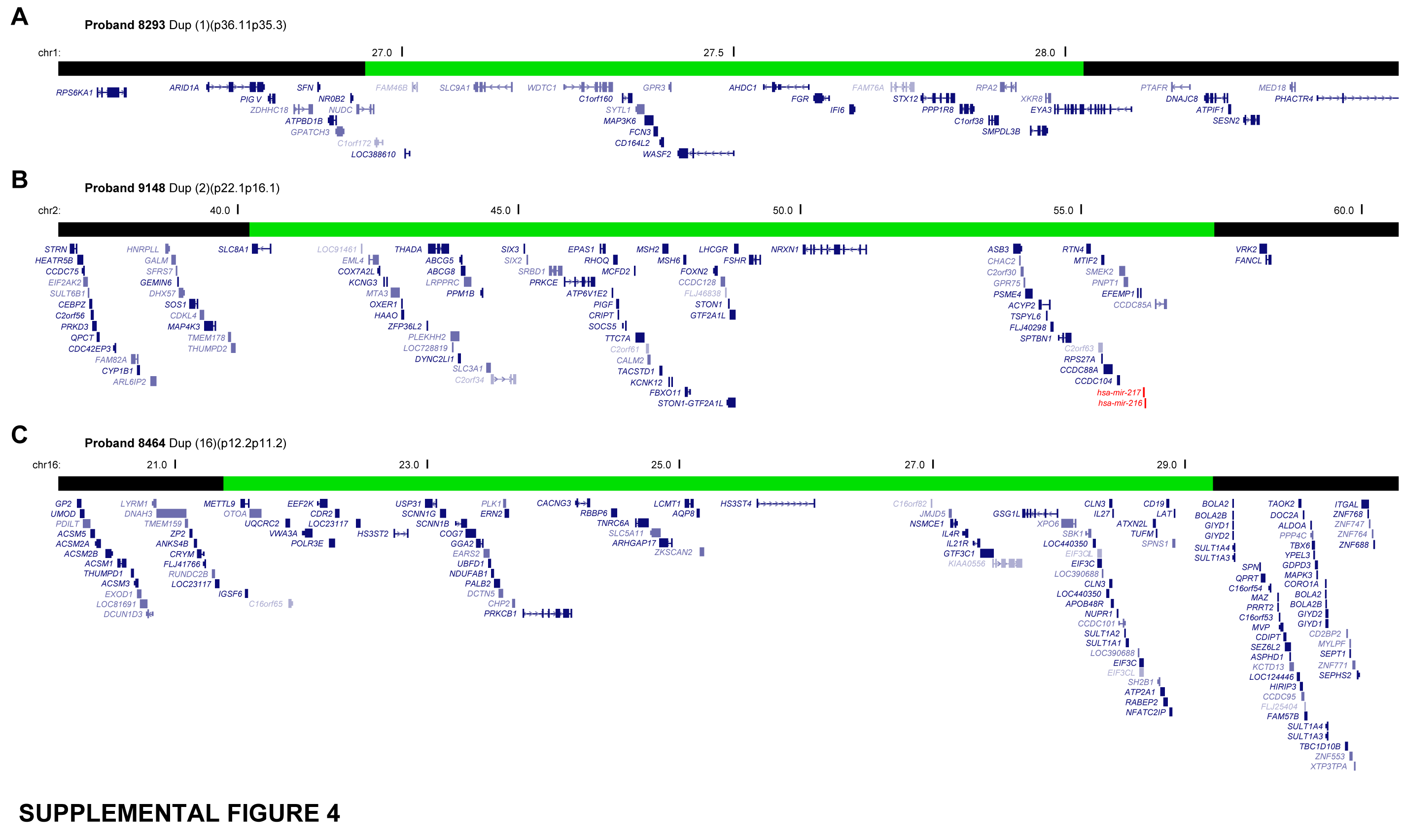

Supplement: Figure S4 — Schematic diagrams showing genes affected by duplications in three probands with ID/MCA. Schematic diagrams of the affected chromosomes showing the location of the duplication on the February 2009 map. The green bars show the location of the duplicated region for the three probands (8293, 9148, 8464) discussed in the text. (0.28 MB TIF) [file pone.0012349.s009.tif]
